# miRNA miR-21 Is Largely Dispensable for Intrathymic T-Cell Development

**DOI:** 10.3389/fimmu.2018.02497

**Published:** 2018-11-05

**Authors:** Heike Kunze-Schumacher, Samantha J. Winter, Esther Imelmann, Andreas Krueger

**Affiliations:** Institute for Molecular Medicine, Goethe University Frankfurt, Frankfurt, Germany

**Keywords:** miRNA, miR-21, thymus, T cells, development, selection, regeneration

## Abstract

Development of T cells in the thymus is tightly controlled to continually produce functional, but not autoreactive, T cells. miRNAs provide a layer of post-transcriptional gene regulation to this process, but the role of many individual miRNAs in T-cell development remains unclear. miR-21 is prominently expressed in immature thymocytes followed by a steep decline in more mature cells. We hypothesized that such a dynamic expression was indicative of a regulatory function in intrathymic T-cell development. To test this hypothesis, we analyzed T-cell development in miR-21-deficient mice at steady state and under competitive conditions in mixed bone-marrow chimeras. We complemented analysis of knock-out animals by employing over-expression *in vivo*. Finally, we assessed miR-21 function in negative selection *in vivo* as well as differentiation in co-cultures. Together, these experiments revealed that miR-21 is largely dispensable for physiologic T-cell development. Given that miR-21 has been implicated in regulation of cellular stress responses, we assessed a potential role of miR-21 in endogenous regeneration of the thymus after sublethal irradiation. Again, miR-21 was completely dispensable in this process. We concluded that, despite prominent and highly dynamic expression in thymocytes, miR-21 expression was not required for physiologic T-cell development or endogenous regeneration.

## Introduction

Development of T cells in the thymus is a tightly controlled process involving the commitment of progenitor cells to the T lineage, generation of a diverse T-cell antigen receptor (TCR) repertoire and selection of largely non-autoreactive T cell clones. Phenotypically, the most immature thymocytes are characterized by the absence of the co-receptors CD4 and CD8 (double-negative, DN), which can be further subdivided into multiple subsets based on dynamic expression of CD117, CD25, and CD44. T-lineage commitment is completed at the CD117^lo^CD25^hi^CD44^+^ DN2b stage upon expression of high levels of the transcription factor Bcl11b (1–4). Somatic recombination of the *Trb, Trg*, and *Trd* TCR gene loci and selection of productive rearrangements is completed at the CD44^−^CD25^+^CD28^+^ DN3b stage. Cells entering the αβT cell lineage acquire expression of both CD4 and CD8 (double-positive, DP), rearrange the *Tra* gene locus and undergo positive and negative selection. Thymocytes then mature into CD4 or CD8 single-positive (SP) cells, where negative selection continues and further maturation occurs prior to egress from the thymus.

T-cell development is controlled by extrinsic factors including cytokines and signals through the TCR. Furthermore, intrinsic factors such as transcriptional programs govern different steps of intrathymic T-cell differentiation have been extensively characterized (5). In contrast, considerably less is known about post-transcriptional regulation of T-cell development, such as by miRNAs (6). Loss of all miRNAs due to deletion of key components of the miRNA processing machinery results in specific defects in T-cell development. Early loss of miRNAs results in profound thymocyte death (7). In addition, a small number of individual miRNAs have been identified to regulate distinct T-lineage developmental stages, including miR-17~92, miR-142, and miR-181a (8–13).

Functional roles of individual miRNAs can only partially explain the effect of loss of all miRNAs observed in T-cell development. In addition, it is possible that some miRNAs exist that display opposing roles in this process. In order to identify miRNAs with a putative function in T-cell development we hypothesized that such miRNAs should be expressed at high levels in at least some thymocyte populations and that such miRNAs should display a pattern of strong dynamic regulation at key developmental checkpoints. miR-21 is prominently expressed in many mammalian tissues (14). In the thymus, expression levels are very high in immature DN thymocytes, followed by a steep decline toward the DP stage and modest re-expression in SP thymocytes (15–17). Expression of miR-21 is induced during T-cell activation and has been reported to support survival of memory T cells and expression of CC chemokine receptor 7 (CCR7) on naïve T cells (18, 19). In addition, it has been proposed that miR-21 promotes PD-1-mediated tolerance by targeting PDCD4 (20). The role of miR-21 in intrathymic T-cell development remains unknown. We hypothesized that high expression levels combined with strong dynamic changes in expression of miR-21 throughout different stages of T-cell development were indicative of a regulatory function in this process. To test this putative function, we analyzed the consequences of miR-21 deletion as well as overexpression in mice *in vivo*. Surprisingly, neither absence nor elevated expression of miR-21 had major effects on T-cell development at steady state or in chimeric mice. Furthermore, endogenous T-cell regeneration remained unaffected by loss of miR-21. We conclude that despite prominent and dynamic expression, miR-21 is dispensable for steady-state and stress-induced T-cell development in mice.

## Results

### Dynamic expression of miR-21 during T-cell development

**Figure 1:**
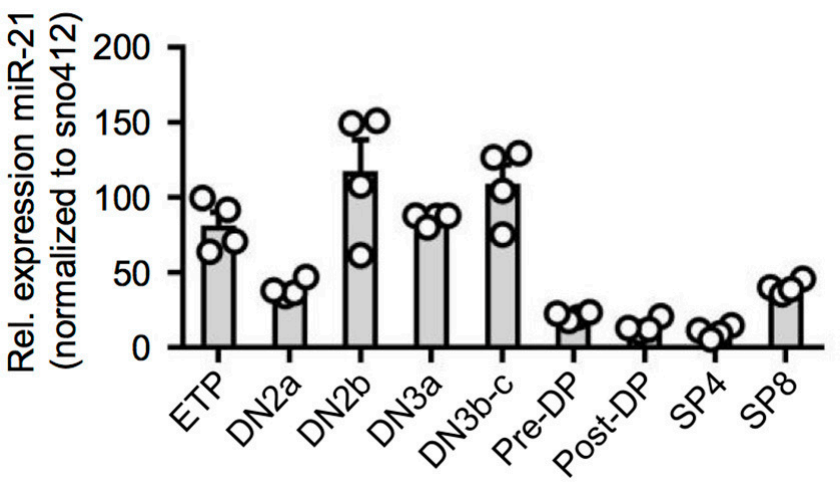
Dynamic expression of miR-21 during T-cell development. Quantitative RT-PCR for miR-21 expression in sorted thymic subsets from WT mice. Expression levels of indicated cell populations were normalized to snoRNA412. Each dot represents one mouse, *n* = 4.

Previous studies determined thymic expression levels of a diverse range of different miRNAs (15–17). For miR-21, thymic expression levels based on miRNA-sequencing were revealed to be high in DN thymocytes accompanied by an abrupt decrease toward the DP stage prior to re-expression in SP T cells. To validate global gene expression data, we sorted thymocyte populations of wildtype (WT) mice starting with ETPs (CD117^hi^CD25^−^CD44^+^), the most immature detectable thymocyte population, toward mature SP T cells and assessed relative expression of miR-21 (Figure 1 and Figure S1). Consistent with previous reports, we found miR-21 to be differentially expressed throughout intrathymic T-cell development. Highest levels of miR-21 were observed in DN thymocytes, especially in DN2b (CD117^lo^CD25^+^CD44^+^) and DN3b (CD25^+^CD44^−^CD28^+^) thymocytes. Our data revealed that lowest levels of miR-21 can be detected in post-selected DP (defined as CD4^+^CD8^+^CD69^+^TCRβ^+^) and mature SP4 T cells (TCRβ^+^CD4^+^). Slightly higher expression was observable in SP8 (TCRβ^+^CD8^+^) confirming previously published data. Together, our findings represent a more detailed analysis about miR-21 expression and validate dynamic expression of miR-21 during T-cell development.

### miR-21 is largely dispensable for steady-state T-cell development in the thymus

**Figure 2:**
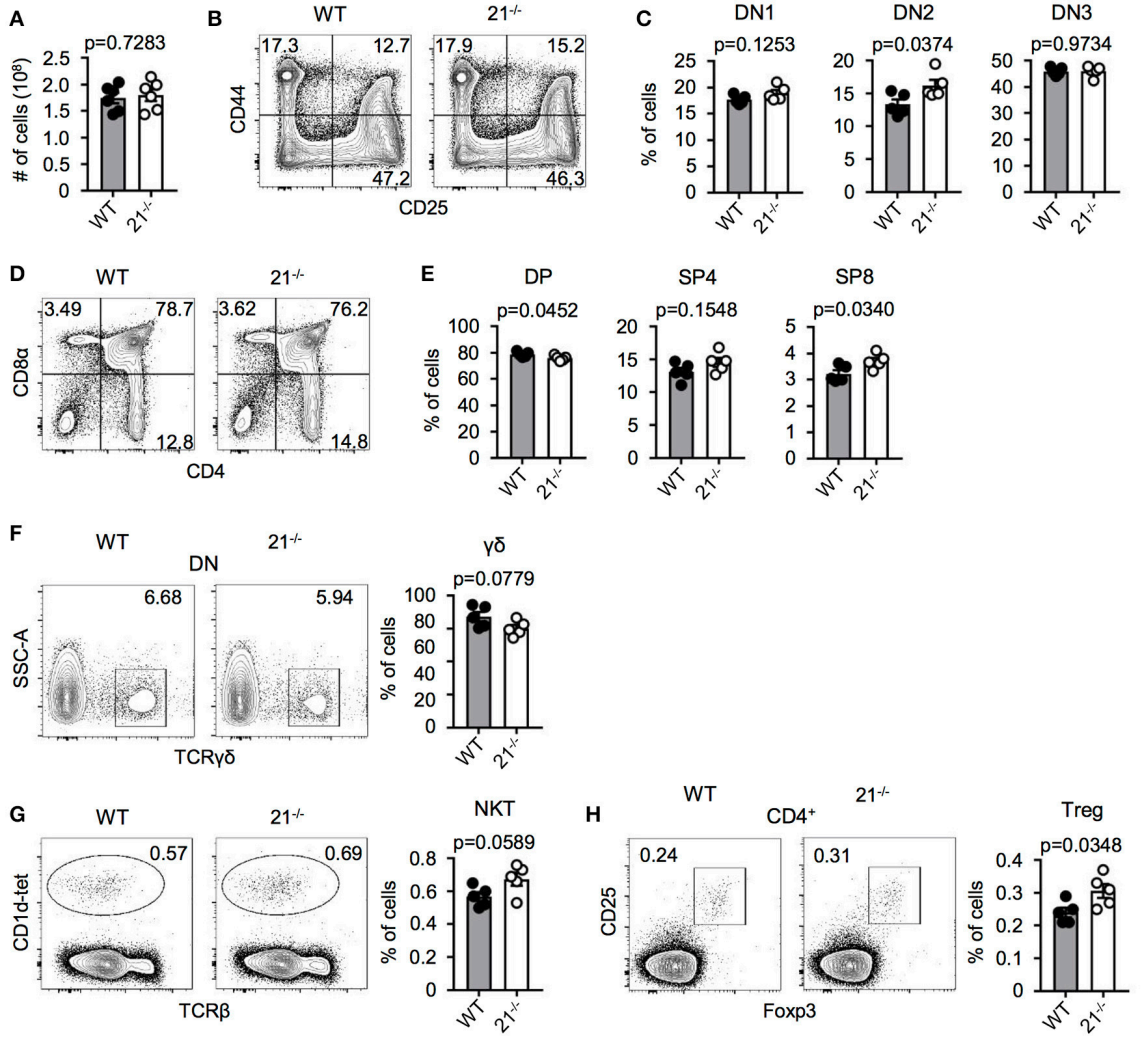
miR-21 is largely dispensable for steady-state T-cell development in the thymus. **(A)** Total cellularity of thymi from WT and miR-21^−/−^ mice, *n* = 6 for each genotype. **(B)** Representative flow cytometric analysis of lineage-depleted thymi from WT and miR-21^−/−^ mice stained with antibodies against CD25 and CD44. Numbers in quadrants represent frequencies. **(C)** Statistical analysis of flow cytometric results shown in **(B)**, *n* = 5. **(D)** Representative flow cytometric analysis of thymi from WT and miR-21^−/−^ mice stained with antibodies against CD4 and CD8α. Numbers in quadrants represent frequencies. **(E)** Statistical analysis of flow cytometric results shown in **(D)**, *n* = 5. **(F)** Representative flow cytometric and statistical analysis of thymi from WT and miR-21^−/−^ mice stained with antibodies against CD4, CD8α and TCRγδ. Numbers in right corners of plots represent frequency, *n* = 5. **(G)** Representative flow cytometric and statistical analysis of thymi from WT and miR-21^−/−^ mice stained with CD1d-tet and antibody against TCRβ. Numbers in right corners of plots represent frequency, *n* = 5. **(H)** Representative flow cytometric and statistical analysis of thymi from WT and miR-21^−/−^ mice stained with antibodies against CD4, CD25 and Foxp3. Numbers in left corners of plots represent frequency, *n* = 5.

In order to test a potential role of miR-21 in intrathymic T-cell development, we first characterized miR-21-deficient mice. Absolute total thymocyte numbers were unaffected by miR-21 (Figure 2A). We then determined early thymocyte subsets in miR-21-sufficient compared to deficient mice and detected a small, but statistically significant increase in the frequency of DN2 thymocytes (Figures 2B,C). When we analyzed late T-cell development in these mice, we observed a slight decrease in frequencies of DP thymocytes (Figures 2D,E) accompanied by increased frequencies of SP T cells. Again, these changes were small. Furthermore, we revealed frequencies of γδ T cells to be similar upon loss of miR-21 (Figure 2F). We and others have shown that miRNAs are essential for the maturation of agonist-selected thymocytes (12, 21–24). To address whether miR-21 might influence the development of invariant natural killer T (iNKT) cell and T regulatory (Treg) cells, we assessed frequencies of these subsets (Figures 2G,H). We detected small but significantly elevated frequencies of Treg cells upon loss of miR-21. This finding is consistent with previous reports that identified miR-21 to modulate Foxp3 transcription factor levels and frequency of circulating Treg cells (25–27). In conclusion, these data suggest that loss of miR-21 does not result in substantial defects in physiological T-cell development.

### Absence of miR-21 does not affect negative selection

**Figure 3:**
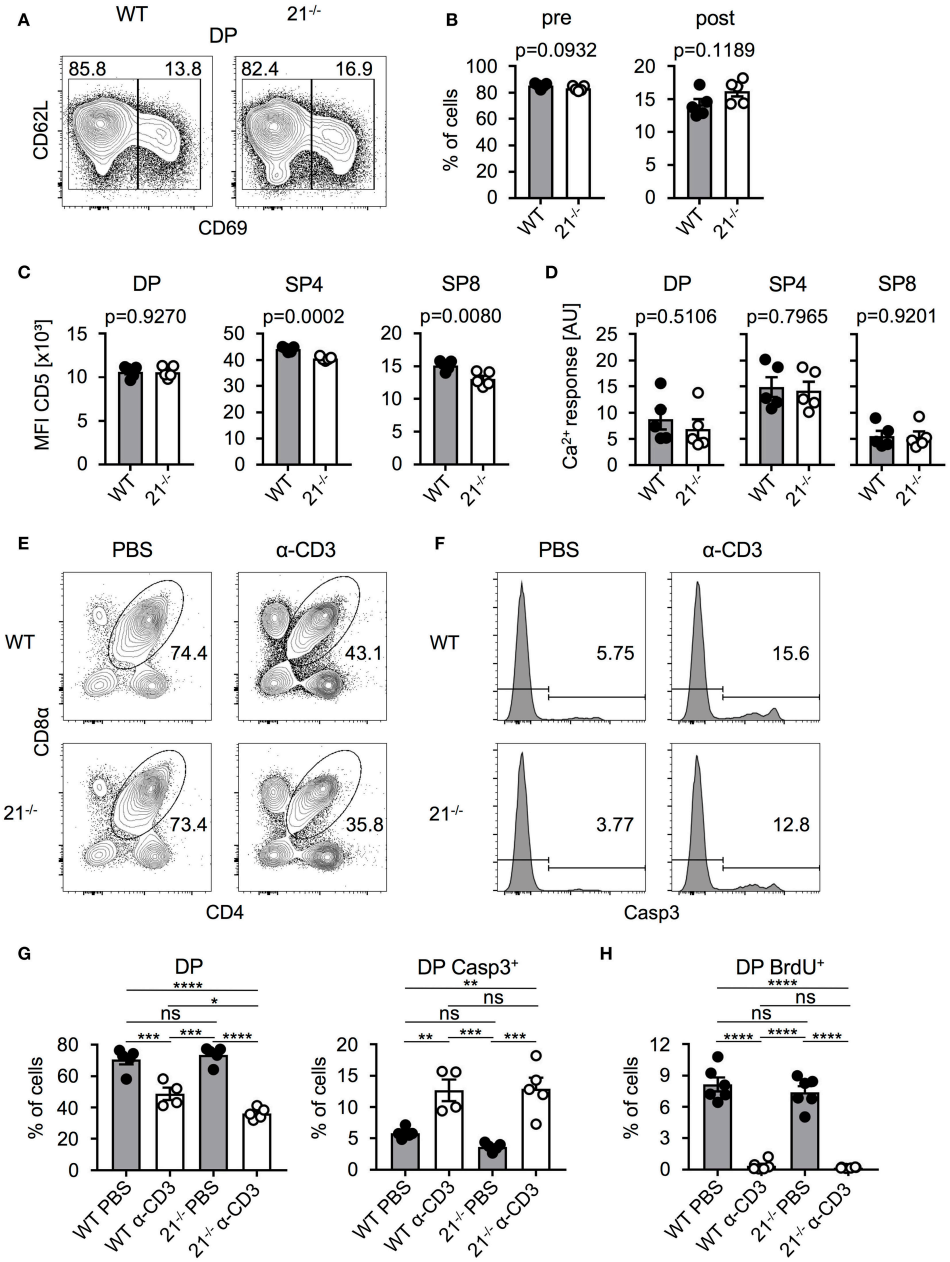
Absence of miR-21 does not affect negative selection. **(A)** Representative flow cytometric analysis of thymi from WT and miR-21^−/−^ mice stained with antibodies against CD4, CD8α, CD69 and CD62L. Numbers in left and right corners represent frequencies of pre- and post-selection DP, respectively. **(B)** Statistical analysis of flow cytometric results shown in **(A)**, *n* = 5. **(C)** Statistical analysis of mean fluorescence intensity of CD5 on DP, SP4 or SP8, *n* = 5. **(D)** Statistical analysis of resulting Ca^2+^-response upon stimulation in DP, SP4 and SP8 thymocytes, *n* = 5. **(E)** Representative flow cytometric analysis of thymi from WT and miR-21^−/−^ mice, 48 h post injection with either PBS or α-CD3, stained with antibodies against CD4 and CD8α. Numbers adjacent to gates represent frequency. **(F)** Histograms display expression of active caspase-3 in DP thymocytes from WT and miR-21^−/−^ mice, 48 h post injection with either PBS or α-CD3, *n* = 4-5 per group. **(G)** Statistical analysis of flow cytometric results shown in **(E,F)**. Analysis of significance were performed using one-way analysis of variance followed by Tukey's test. Indicated significant results are representative of latter test. **(H)** Statistical analysis of BrdU^+^ DP thymocytes of thymi from WT and miR-21^−/−^ mice, 48 h post injection with either PBS or α-CD3, *n* = 6 for each genotype. Pooled data of two independent experiments.

Based on our previous finding that frequencies of DP thymocytes were significantly reduced in miR-21-deficient mice, we next determined whether frequencies of pre- and post-selected DP thymocytes were skewed in miR-21^−/−^ mice. To this end, we identified these populations based on their surface expression of CD69 and CD62L and detected no alterations in the absence of miR-21 compared to their WT counterparts (Figures 3A,B). We then asked whether miR-21 modulates selection processes. To test this, we first analyzed surface expression of CD5 as an indicator of TCR signal strength in DP, SP4, and SP8 thymocytes of miR-21^−/−^ mice. We detected comparable CD5 expression of DP thymocytes between WT and miR-21^−/−^ mice, whereas SP4 and SP8 T cells of miR-21^−/−^ mice revealed diminished expression levels (Figure 3C). We then tested a potential role of miR-21 in modulating TCR signal strength by studying Ca^2+^-flux upon stimulation with anti-CD3 antibody. We did not detect any significant differences in the ability of miR-21-deficient DP or SP thymocytes to respond to anti-CD3 stimulation both in terms of peak response (Figure 3D) and overall kinetics (Figure S4A). Furthermore, we evaluated whether loss of miR-21 leads to altered viability of SP thymocytes by studying *ex vivo* survival of these cells (Figure S5). We did not observe significant differences in early apoptotic cells and only a mild decrease in the frequency of late apoptotic SP8 cells after 48 h in culture, indicating that survival of SP thymocytes is largely unaffected by loss of miR-21. To further evaluate whether a functional defect in DP thymocytes could exist that modulates negative selection, we adapted an *in vivo* TCR stimulation approach (28). First, we injected WT or miR-21^−/−^ mice with a single dose of anti-CD3 (or PBS as a control) to stimulate TCR signaling. After 48 h, we assessed frequencies of thymocytes and performed staining for activated caspase-3 to examine early apoptosis (Figures 3E-G). Thymi of control mice contained normal frequencies of DP thymocytes, whereas thymi derived from either WT or miR-21^−/−^ anti-CD3-treated mice showed significantly lower frequencies. Frequencies of DP thymocytes were slightly reduced in miR-21-deficient mice upon treatment with anti-CD3 compared to their WT counterparts, which could be due to increased apoptosis levels in miR-21-deficient mice as previously described (29). However, analysis of activated caspase-3 showed no significant differences in apoptosis between WT and miR-21-deficient DP thymocytes after treatment. In addition, we determined thymic cellularity of PBS- or anti-CD3-treated WT and miR-21^−/−^ mice and found absolute total thymocyte numbers to be unaffected by miR-21 upon treatment (Figure S4B). Finally, we monitored proliferation in this assay by administration of a single BrdU pulse (Figure 3H). We found similar frequencies of BrdU incorporation in DP thymocytes of WT or miR-21^−/−^ controls treated with PBS, indicating equal rates of proliferation at steady state. Virtually no BrdU incorporation was detected in thymocytes after anti-CD3-stimulation, which is consistent with the experimental setup to monitor negative selection. Thus, we concluded that miR-21 is largely dispensable during thymic selection.

### Physiological peripheral lymphoid cell frequencies in miR-21^−/−^ mice

**Figure 4:**
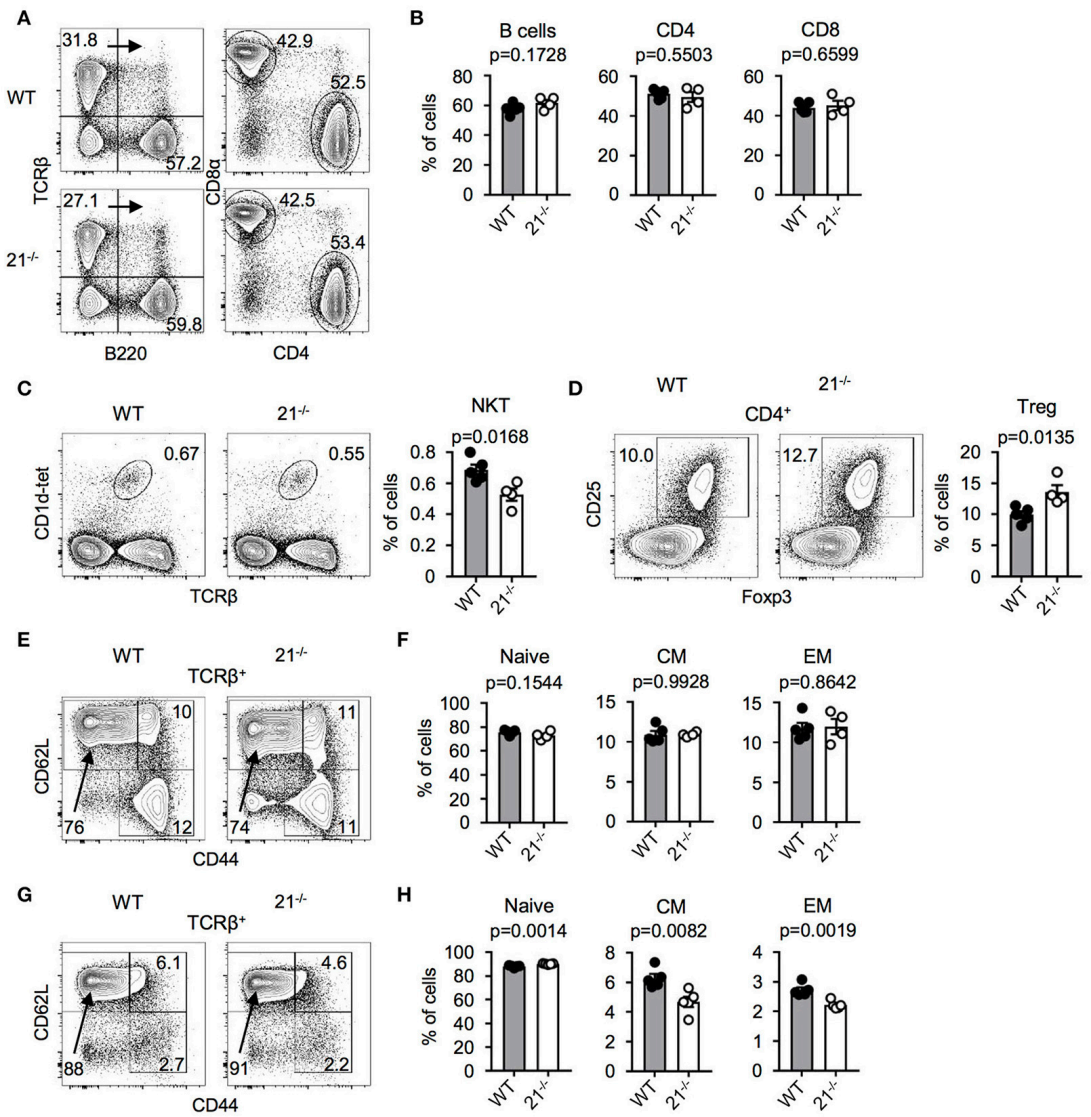
Physiological peripheral lymphoid cell frequencies in miR-21^−/−^ mice. **(A)** Representative flow cytometric analysis and gating strategy of spleen from WT and miR-21^−/−^ mice stained with antibodies against B220, TCRβ, CD4, and CD8α. Numbers in quadrants or adjacent to gates represent frequencies. **(B)** Statistical analysis of flow cytometric results shown in **(A)**, *n* = 4-5. **(C)** Representative flow cytometric and statistical analysis of spleen from WT and miR-21^−/−^ mice stained with CD1d-tet and antibody against TCRβ. Numbers in right corners of plots represent frequency, *n* = 4-5. **(D)** Representative flow cytometric and statistical analysis of spleen from WT and miR-21^−/−^ mice stained with antibodies against CD4, CD25, and Foxp3. Numbers in left corners of plots represent frequency, *n* = 4-5. **(E)** Representative flow cytometric analysis of spleen from WT and miR-21^−/−^ mice stained with antibodies against TCRβ, CD44, and CD62L to identify naïve (CD44^−^CD62L^+^), central memory (CD44^+^CD62L^+^) and effector memory (CD44^+^CD62L^−^) T-cell subsets. Numbers in or adjacent to gates represent frequency. **(F)** Statistical analysis of flow cytometric results shown in **(E)**, *n* = 4-5. **(G)** Representative flow cytometric analysis of LNs from WT and miR-21^−/−^ mice stained with antibodies against TCRβ, CD44, and CD62L to identify naïve (CD44^−^CD62L^+^), central memory (CD44^+^CD62L^+^) and effector memory (CD44^+^CD62L^−^) T-cell subsets. Numbers in or adjacent to gates represent frequency. **(H)** Statistical analysis of flow cytometric results shown in **(G)**, *n* = 5.

Next, we addressed the question whether miR-21 is important for peripheral lymphoid cell subsets. Therefore, we analyzed spleen and lymph nodes (LNs) of miR-21-deficient mice and found miR-21 to be dispensable for peripheral B cells and T cell subsets (Figures 4A,B). Frequencies of agonist-selected iNKT and Treg cells in the periphery showed small differences (Figures 4C,D) that recapitulated our findings observed in the thymus. Furthermore, we analyzed the distribution of naïve, central memory, and effector memory T-cell subsets in spleen (Figures 4E,F) and LN (Figures 4G,H) and detected small changes in LN of miR-21-deficient mice. To further evaluate the role of miR-21, we analyzed pre-thymic progenitors in the bone marrow (BM). We detected similar frequencies of hematopoietic stem cells (HSCs), multipotent progenitors (MPPs), and common lymphoid progenitors (CLPs) upon loss of miR-21 (Figure S2), thus arguing against a pivotal role of miR-21 in the periphery.

### miR-21-deficiency or overexpression do not alter T-cell development in BM chimeras

**Figure 5:**
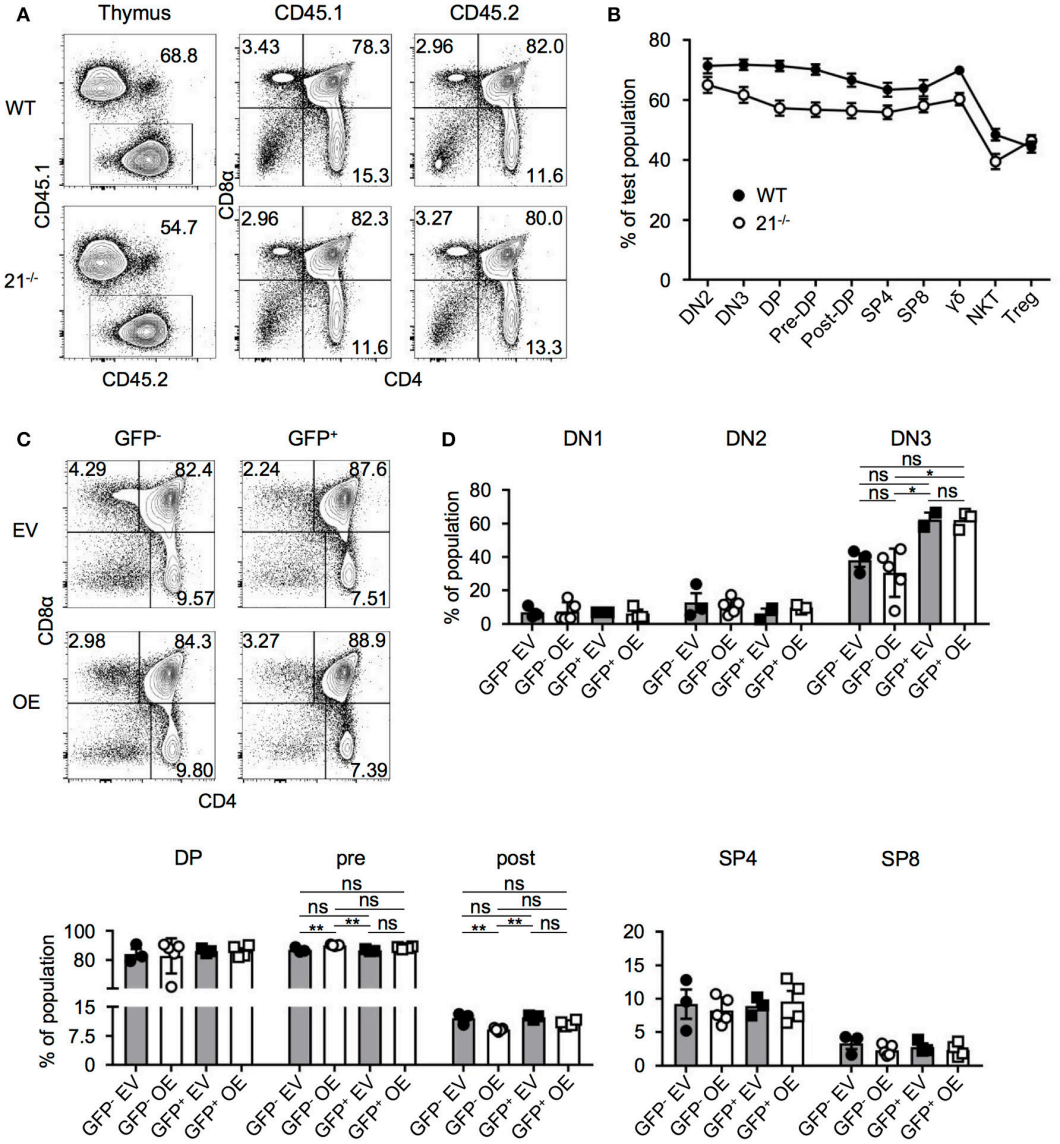
miR-21-deficiency or overexpression do not alter T-cell development in BM chimeras. **(A)** Representative flow cytometric analysis of competitive BM chimeras 8 weeks after transfer, CD45.1: competitor population; CD45.2: test population (WT control or miR-21^−/−^). Thymi were stained with antibodies against CD45.1, CD45.2, CD4, and CD8α. Numbers adjacent to gates and in quadrants represent frequencies. **(B)** Comparative analysis of thymocyte subpopulations in CD45.2 test populations (WT control or miR-21^−/−^) of competitive chimeras, *n* = 9 per group. **(C)** Representative flow cytometric analysis of overexpression chimeras 8 weeks after transfer. Thymi were stained with antibodies against CD4 and CD8α. Left panels display distribution of GFP^−^ (untransduced) thymocytes in EV (empty vector) control or OE (miR-21-overexpression) chimeras. Right panels indicate GFP^+^ (transduced) thymocytes. Numbers in quadrants represent frequencies. **(D)** Statistical analysis of flow cytometric results of DN1, DN2, DN3, DP, pre- and post-selection DP, SP4, and SP8 populations from GFP^−^ EV, GFP^−^ OE, GFP^+^ EV, GFP^+^ OE thymocyte subsets indicated as frequencies, inter alia shown in **(C)**, *n* = 2-5. Analysis of significance were performed using one-way analysis of variance followed by Tukey's test. Indicated significant results are representative of latter test.

Competitive BM chimera experiments are able to reveal cell-intrinsic defects in development, which at steady state, may be masked by compensatory effects (8, 30–33). Thus, we asked whether a possible cell intrinsic advantage or disadvantage of miR-21-deficient BM-derived cells could occur. Lethally irradiated WT recipients were reconstituted with a 1:1 mixture of BM from WT (CD45.1) and BM cells from miR-21-deficient (CD45.2) or control mice (CD45.2). After 8 weeks, we analyzed thymi of these chimeric mice and identified miR-21^−/−^-derived BM cells to compete in a similar manner as the respective WT controls (Figures 5A,B). Detailed analysis of frequencies of DP, SP4, and SP8 suggested no aberrant effect of miR-21-deficient BM cells. Accordingly, we detected comparable frequencies of these T-cell subsets in total thymus, CD45.1–or CD45.2-derived cells (Figure 5A). For DN thymocytes, we found small differences in their reconstitution levels. DN2 thymocytes of miR-21^−/−^ origin competed in a similar manner as the provided WT control and miR-21^−/−^-derived DN3 thymocytes as well as DP thymocytes were detected in slightly decreased frequencies indicated by a decline in contribution. For later stages of T-cell development, this effect was absent as indicated by similar reconstitution rates of miR-21^−/−^ and WT control BM. Notably, Treg cells derived from miR-21-deficient BM cells displayed a beneficial reconstitution compared to their control WT counterparts. This observation can be explained by increased radioresistance of Treg cells (34–36). Therefore, host-derived Treg cells are not fully eliminated and might contribute to this skewed reconstitution.

Based on our findings that loss of miR-21 does not result in aberrant T-cell development, we next aimed to elucidate whether overexpression of miR-21 controls thymopoiesis. We overexpressed miR-21 (and an empty vector control) retrovirally in lineage-depleted BM cells prior to reconstitution of WT mice. Both vectors encoded enhanced GFP (eGFP) as a reporter gene. After 8 weeks, thymi were harvested and analyzed for the development of T cells (Figures 5C,D). First, we assessed frequencies of DP, SP4, and SP8 cells in either GFP^−^ (untransduced) or GFP^+^ cells (transduced) of control and miR-21-overexpressing thymi and detected no significant alterations. In addition, we studied early thymocyte subsets and found no differences in frequencies of DN1 and DN2 upon overexpression (Figure 5D). For DN3 thymocytes, a slight increase in their frequency upon overexpression was observable when compared to their untransduced (GFP^−^) counterparts. However, when compared to the empty vector control, no significant difference was detectable. Similarly, frequencies of pre- and post-selection DP thymocytes were comparable to their control counterparts upon overexpression (Figure 5D). Thus, we concluded that ectopic elevation of miR-21 levels does not abrogate intrathymic T-cell development.

### T-lineage and alternative lineage fate decisions are mostly unaltered in the absence of miR-21

**Figure 6:**
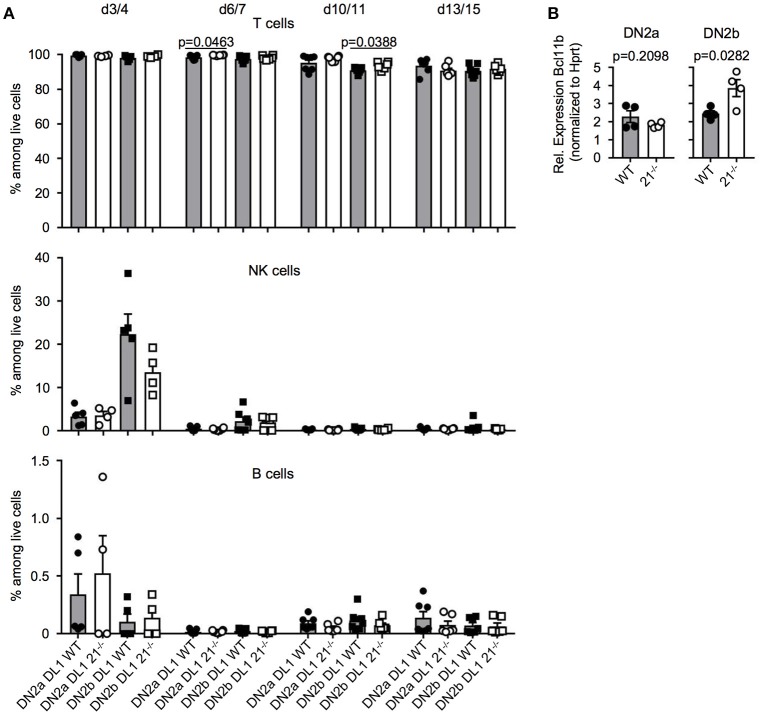
T-lineage and alternative lineage fate decisions are mostly unaltered in the absence of miR-21. **(A)** Sorted DN2a and DN2b cells were cultured on OP9-DL1 cells for up to 15 days. Generation of T, NK, and B cells was assessed by flow cytometry at indicated periods of time. Pooled data of two independent experiment. Each dot represents one mouse, *n* = 4-7 per group. **(B)** Analysis of expression of *Bcl11b* in sorted DN2a and b thymocytes of WT or miR-21^−/−^ mice by quantitative RT-PCR. Expression levels were normalized to *Hprt*. Each dot represents one mouse, *n* = 4.

In a next step, we tested whether loss of miR-21 may result in altered potential to generate lymphocytes. We utilized the OP9-DL1 and OP9-GFP system to monitor T, B, and NK lineage differentiation *in vitro*. In general, T-lineage commitment is finalized with expression of the transcription factor Bcl11b at the DN2 stage. This stage can be further subdivided, based on the expression of CD117 into DN2a and DN2b. DN2a thymocytes have not fully undergone T-lineage fate decision and still contain non-T cell options such as NK cell potential, whereas the DN2b stage reflects the point of no return in regards to T-lineage fate decision (37). For this reason, we sorted DN2a and DN2b cells from either WT or miR-21^−/−^ mice and cultured those on OP9-DL1 and OP9-GFP cells for up to 15 days. We determined the frequencies of generated T, NK and B cells over time and observed no detectable changes compared to WT on OP9-DL1 (Figure 6A) or on OP9-GFP cells (Figure S3). Given the critical role of the transcription factor Bcl11b at this stage, we assessed its relative abundance in DN2a and DN2b of miR-21-sufficient and deficient mice. We identified elevated Bcl11b levels upon loss of miR-21 in DN2b thymocytes (Figure 6B) indicating a preference of miR-21 in promoting alternative lineage fate decisions. However, *in vitro* differentiation indicated that physiological levels of miR-21 are sufficient to maintain adequate levels of Bcl11b. Thus, we concluded that loss of miR-21 does not result in detectable changes regarding lineage potential of DN2 thymocytes.

### Loss of miR-21 does not affect endogenous T-cell regeneration

**Figure 7:**
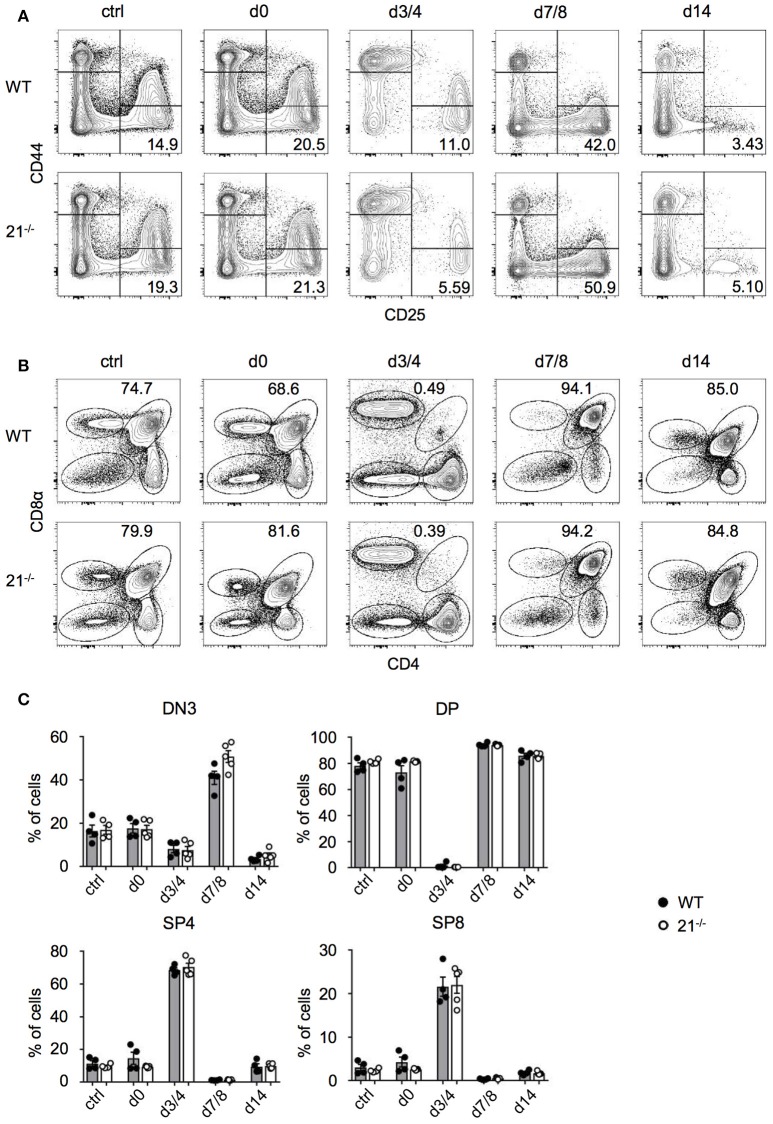
Loss of miR-21 does not affect endogenous T-cell regeneration. **(A)** Representative flow cytometric analysis of thymi from WT and miR-21^−/−^ mice stained with antibodies against CD25 and CD44. Numbers in right bottom quadrants represent frequencies of DN3 thymocytes. Regeneration of DN thymocytes was assessed at indicated periods of time in non-irradiated mice or post sublethal irradiation. Pooled data of two independent experiments. **(B)** Representative flow cytometric analysis of thymi from WT and miR-21^−/−^ mice stained with antibodies against CD4 and CD8α. Numbers adjacent to right gate represent frequencies of DP thymocytes. Regeneration of DP, SP4, and SP8 thymocytes was assessed at indicated periods of time in non-irradiated mice or post sublethal irradiation. Pooled data of two independent experiments. **(C)** Statistical analysis of flow cytometric results shown in **(A,B)**. Each dot represents one mouse, *n* = 4-5 per genotype. Statistical analysis were performed using unpaired Mann-Whitney test.

Having defined the dispensable role of miR-21 in steady-state thymus and periphery, we next addressed whether miR-21 is contributing to stress regulated responses. We adopted an approach from Dudakov et al. (38) using sublethal total body irradiation to induce thymic stress and subsequent endogenous regeneration (Figures 7A-C). We assessed frequencies of DN and DP thymocytes every third to fourth day. As controls, we used non-irradiated mice as well as irradiated mice (both WT and miR-21^−/−^), which were immediately sacrificed upon irradiation. At day 3/4 post irradiation, we detected nearly complete absence of DP thymocytes in WT and miR-21^−/−^ thymi, whereas frequencies of SP4 and SP8 cells were highest at this time point. This pattern was inversed at day 7/8. We observed profound changes in levels of DN thymocytes as indicated by the expression of CD25 and CD44 over time. These changes were largely dependent on the loss of DP cells. In light of this, frequencies of DN3 thymocytes reached highest levels 7 days post irradiation in WT and miR-21-deficient mice, but after 14 days, frequencies declined again. We did not observe any statistically significant differences between WT and miR-21-deficient mice. Thus, we concluded that miR-21 does not contribute to endogenous regeneration of the thymus after sublethal irradiation.

## Discussion

We confirmed miRNA expression profiling showing that miR-21 is prominently expressed in thymocytes and is subject to substantial changes in expression levels during T-cell development (15–17). Hence, we hypothesized that defined expression levels of miR-21 were required to sustain physiologic T-cell development. We tested this hypothesis using a variety of approaches, including analysis of miR-21-deficient mice at steady state, competitive BM chimeras in the contexts of miR-21 deficiency and over-expression, as well as *in vitro* differentiation. When comparing miR-21-deficient and sufficient mice at steady state, we observed some statistically significant differences in population sizes for DN2 cells as well as Treg cells, reduced expression levels of CD5, a surrogate marker for TCR signal strength and elevated levels of Bcl11b in DN2b cells. However, throughout the magnitude of these differences were minor and could not be validated in more functional assays, such as anti-CD3-mediated induction of negative selection or competitive BM chimeras. Together, we concluded that miR-21 is largely dispensable for intrathymic T-cell development.

Despite being prominently expressed in multiple organ systems, including immune system, liver and cardiovascular system (14), information on the physiologic role of miR-21 remains scarce. miR-21 has been implicated as negative regulator of T-cell activation, survival factor for memory cells as well as downstream effector of PD-1 signaling (18–20, 39).

It has been suggested that miR-21 plays a role in multiple pathological conditions. Thus, treatment of mice with miR-21 antagonists was able to reduce pressure induced cardiac failure and similar antagomiRs were protective in autoimmune encephalomyelitis, a mouse model of multiple sclerosis (40, 41). miR-21 is better characterized as an oncogene in a variety of malignancies. Overexpression of miR-21 in mice results in the formation of pre-B-cell lymphomas, which regress once expression of miR-21 is shut off (29). Furthermore, overexpression of miR-21 promoted non-small-cell lung cancer, whereas loss of miR-21 suppressed development of tumors (42, 43). Consistent with our finding that miR-21 is essentially dispensable for steady-state T-cell development, these studies suggest that the role of miR-21 appears to be largely restricted to cellular stress or pathological settings, most prominently cancer.

Based on this assumption, we assessed a potential role of miR-21 in a model of thymic regeneration. Endogenous regeneration is critical for restoration of immune competence after stress or pre-conditioning for hematopoietic stem cell transplantation (44). However, also in this model miR-21 turned out to be completely expendable.

What might be the reason underlying the mild or absent phenotype in steady-state miR-21-deficient mice? Notably, the most prominent functions of miR-21 have been discovered using pharmacological inhibition of miR-21 using antagonists (40, 41). In contrast, constitutive loss of miR-21 appears to be only functional in the context of malignancy (42, 43, 45). This discrepancy suggests that in the unperturbed organism, constitutive loss of miR-21 might be compensated for by a yet to be identified mechanism (46). Recently, it has been demonstrated that, in contrast to tumor cell lines, virtually no de-repression of miR-21 targets can be observed in healthy mouse liver from miR-21-deficient mice or upon treatment with miR-21 inhibitors (47). This lack of target recognition by miR-21 was not due to specific characteristics of the seed region, but correlated with lack of association with polysomes, when compared to other miRNAs. Thus, in healthy cells, miR-21 is selectively absent from translationally very active mRNA. This finding helps to explain our finding that, despite high levels of expression and dynamic changes throughout development, miR-21 does not contribute to physiologic T-cell development and thymic regeneration.

Nevertheless, the underlying molecular mechanism that selectively prevents miR-21 to actively target actively translated mRNA remains unclear. So far, expression of miR-21 has almost exclusively been associated with pathology. However, ultimately, it appears unlikely that evolution should conserve an exclusively pathogenic molecule, so that the quest to identify the physiologic function of miR-21 remains ongoing.

## Materials and methods

### Mice

miR-21 knockout mice (B6;129S6-Mir21a^tm1Yoli^/J; termed miR-21^−/−^ mice throughout this article) were purchased from The Jackson Laboratory (Bar Harbor, ME). C57BL/6N mice (CD45.2) were obtained from Charles River Laboratories, Germany. B6.SJL-*Ptprc*^*a*^*Pepc*^*b*^*/BoyJ* mice (CD45.1) and C57BL/6N x *Ptprc*^*a*^*Pepc*^*b*^*/BoyJ* F1 mice (CD45.1/CD45.2 heterozygous) were bred at the ZFE, Goethe University Frankfurt. All mice were used between 4 and 20 weeks of age and maintained under specific pathogen-free conditions.

### Ethics statement

All animal experiments were performed in accordance with local and institutional guidelines and have been approved by the Regierungspräsidium Darmstadt, Abteilung Veterinärwesen.

### Flow cytometry and cell sorting

Monoclonal antibodies specific for B220 (RA3-6B2), CCR7 (4B12), CD4 (GK1.5), CD5 (53-7.3), CD8α (53-6.7), CD24 (M1/69), CD25 (PC61.5), CD28 (E18), CD44 (IM7), CD45.1 (A20), CD45.2 (104), CD62L (MEL-14), CD69 (H1.2F3), CD117 (2B8), CD127 (SB/199), CD135 (A2F10), Foxp3 (MF23), NK1.1 (PK136), TCRβ (H57-597), TCRγδ (GL3), Sca-1 (D7) were used as AmCyan, Brilliant Violet 510 (BV510), BV421, Pacific Blue (PB), eFluor450, fluorescein isothiocyanate (FITC), Alexa488, Alexa647, phycoerythrin (PE), peridinin chlorophyll protein-Cy5.5 (PerCP-Cy5.5), PE-Cy7, APC, APC-Cy7 and were purchased from eBioscience, BD Biosciences, or Biolegend. Allophycocyanin (APC)-conjugated CD1d/PBS-57 (αGalCer analog) loaded and unloaded tetramers were provided by the NIH Tetramer Core Facility. Cells were acquired using a BD FACSCanto II and data was analyzed using FlowJo software (Tree Star). Lin^−^ cells were isolated from total BM by staining cell suspensions obtained from the femur and tibia with a lineage-specific antibody cocktail followed by magnetic bead depletion using the Lineage Cell Depletion Kit as per manufacturer's instructions (Miltenyi Biotec). DN thymocytes were isolated from total thymocytes by staining cell suspensions with anti-CD4 (RL1.72) and anti-CD8 (M31), followed by lysis with Low Tox-M Rabbit Complement (Cedarlane Laboratories) and subsequent density gradient centrifugation with Lympholyte-M (Cedarlane Laboratories). Cells were sorted using a FACS Aria II cell sorter.

### Cell preparations

Thymus, spleen and inguinal lymph nodes were meshed through a 70 μm cell strainer (Corning) to obtain single-cell suspensions. For spleen, red blood cells (RBCs) were depleted using Qiagen RBC Lysis Solution according to manufacturer's instructions.

### Construction of miRNA expression vectors

The retroviral construct MDH1-PGK-GFP_2.0-miR-21 was generated to achieve stable overexpression of miR-21. For this reason, the 273-bp miRNA gene segment containing the miR-21 hairpin was amplified and introduced via *Xho*I and *Eco*RI in the 3' LTR under control of the human H1 promoter of the empty vector MDH1-PGK-GFP_2.0. The empty vector was a gift from Chang-Zheng Chen (Addgene plasmid # 11375) and served as a control. For both vectors, eGFP is encoded as a reporter gene under control of the PGK promoter and served to determine transduction efficiency.

### Retroviral transduction

For production of miR-21-expressing virus particles, HEK293T cells were transfected with pCL-Eco (co-expressing gag, pol and env derived from murine leukema virus) and MDH1-PGK-GFP_2.0-miR-21 or the empty vector control for 8 h using a calcium phosphate transfection kit (Takara). Subsequently, medium was exchanged and supernatant containing retroviral particles was harvested 24, 48, and 72 h after transfection. For transduction of lineage-depleted BM, cells were cultured overnight in minimum essential medium (Gibco) containing 20% FCS (GE Healthcare) in the presence of mouse SCF (50 ng/mL), IL-7 (25 ng/mL), Flt3-L (25 ng/mL), and IL-6 (20 ng/mL) (all obtained from Peprotech) at a density of 1 × 10^6^ cells/mL in a 24-well plate (Sarstedt). On day 2 and 3, cells were transduced with respective viruses supplemented with polybrene (8 μg/mL; Sigma-Aldrich) using spin infection (700 *g*, 1.5 h, room temperature) and subsequent incubation for 4 h in a 37°C incubator before replacing with fresh medium containing cytokines. The following day, 5 × 10^5^ cells were injected intravenously into recipients 4 h after lethal irradiation (9 Gy). Mice were given antibiotics in the drinking water for 2 weeks and analyzed after 8 weeks.

### Competitive BM chimeras

Donor BM cells from WT (CD45.1, competitor), WT (CD45.2, test) and miR-21^−/−^ (CD45.2, test) were prepared as described above. Recipient CD45.1/CD45.2 mice were lethally irradiated (9 Gy) and reconstituted intravenously with mixtures of competitor and test cells in a 1:1 ratio 4 h after irradiation (a total of 2 × 10^6^ cells per mouse). Mice were treated with antibiotics in the drinking water for 2 weeks and analyzed after 8 weeks.

### OP9 cocultures

OP9-DL1/OP9-GFP co-culture assays were essentially performed as described previously (48). Sorted precursors (DN2a and DN2b) were plated onto subconfluent OP9 BM stromal cells expressing either the Notch ligand Delta-like ligand 1 (OP9-DL1) or GFP (OP9-GFP) as a control in a 24-well plate. Cocultures were performed in the presence of 10 ng/mL SCF, 5 ng/mL Flt3-L, and 1 ng/mL IL-7 for OP9-DL1 T cell differentiation and 5 ng/mL IL-7 for OP9-GFP cultures (all obtained from Peprotech). After 4 days, half of the culture medium was replaced with fresh medium and at day 7 of differentiation thymocytes were harvested and separated from contaminating OP9 cells by filtering the cocultured cells through a 50 μm filter (Sysmex) prior to seed onto fresh OP9 monolayers. Lineage differentiation was monitored for up to 15 days. Discrimination of dead cells were performed using 7-amino-actinomycin D according to the manufacturer's instructions.

### Quantitative real-time PCR (qRT-PCR)

Total RNA from different sorted mouse T cells was prepared using the miRNeasy kit (Qiagen) as recommended by the manufacturer. Expression of miR-21 was assessed by quantitative qRT-PCR using miRNA specific looped reverse transcriptase primers and TaqMan probes for hsa-miR-21 (Applied Biosystems, Taqman miRNA Assay ID 00397). Relative Expression was calculated as % expression of snoRNA412 as house-keeping genes using the Δ cycle threshold method (Applied Biosystems, ID 001243).

Expression of Bcl11b was determined by reverse transcription using M-MLV Reverse Transcriptase (Promega) and random hexamer Oligo(dT)12-18 primers (Progmega) according to the manufacturer's protocol. Quantitative RT-PCR analysis of Bcl11b expression was performed using the respective Bcl11b TaqMan probe (Applied Biosystems, Mm01332818_m1). Fold differences were calculated using the Δ cycle threshold method normalized to *Hprt* as housekeeping gene (Applied Biosystems, Mm00446968_m1).

All reactions were performed as triplicates on a Quantstudio 3 (Applied Biosystems).

### Thymus regeneration

WT or miR-21^−/−^ mice were sublethally irradiated (5,5 Gy) and thymi were harvested at days 0, 3, 7, and 14 post irradiation. Cells were stained with monoclonal antibodies against lineage markers (CD4, CD8, CD25, CD44, and CD117) followed by viability staining and were analyzed by flow cytometry.

### *In vivo* T-cell receptor stimulation

Mice, WT or miR-21^−/−^, were injected with 200 μg purified anti-mouse CD3 (17A2, Biolegend). After 48 h, thymi were harvested, stained with monoclonal antibodies against lineage markers followed by Zombie Aqua™ (Biolegend) staining to exclude dead cells. Cells were fixed using formaldehyde (4%) and permeabilized prior to caspase-3 staining (Asp175, CST) followed by staining with secondary donkey anti-rabbit-Alexa647 (Poly4064, Biolegend) and flow cytometric analysis. For BrdU incorporation, mice were injected i.v. (2 mg/mouse) 3 h before analysis. Staining for BrdU was performed according to the manufacturer's instructions using a FITC anti-BrdU antibody (Biolegend, clone: 3D4) after fixation, permeabilization (BD Biosciences) and DNase-treatment of the cells (Sigma-Aldrich, 30 μg).

### Apoptosis detection assay

Single-cell suspensions of thymocytes from WT and miR-21^−/−^ mice were incubated for up to 48 h cells in minimum essential medium (Gibco) containing 20% FCS (GE Healthcare). After 0, 24 and 48 h, cells were stained with monoclonal antibodies against CD4 and CD8 followed by Annexin V detection and staining with PI according to manufacturer's kit instructions (Biolegend).

### Measurement of intracellular Ca^2+^-flux in thymocytes

Influx of Ca^2+^ was performed as previously described (12) with minor modifications. Briefly, 5 × 10^6^ thymocytes from CD45.1 WT mice were mixed with an equal amount of thymocytes from miR-21^−/−^ mice and incubated for 1 h in 1 mL Ca^2+^- and Mg^2+^-free Hanks' PBS (HBSS, pH 7.4) (Sigma-Aldrich) at room temperature. Cells were washed and loaded with Fluo-4 (3 μM) and FuraRed (6 μM) dyes (both Invitrogen) and incubated at 37°C in Ca^2+^- and Mg^2+^-sufficient HBSS (Gibco). After 45 min, cells were simultaneously stained for CD45.1, CD45.2, CD4, and CD8α and stimulated with 100 μg purified anti-mouse CD3 (17A2, Biolegend) for 30 min on ice. Subsequently, cells were rested at 37°C for 30 min. For flow cytometric analysis, a baseline for 30 s was recorded prior to addition of 12 μg goat anti-rat antibody (Jackson ImmunoResearch). Data acquisition was performed for additional 210 s. Finally, 2 μg ionomycin (Sigma-Aldrich) were added as positive control. For data analysis, median peak height resulting from anti-CD3-stimulation was determined.

### Statistical analysis

All analysis was performed using GraphPad Prism software (version 7). Data are represented as mean plus or minus SEM and *P* < 0.05 was considered as significant. In all figures, ^*^P < 0.05; ^**^P < 0.01; ^***^P < 0.001; ^****^P < 0.0001; ns, not significant. Analysis of significance between 2 groups of mice was performed using unpaired *t-*tests unless otherwise specified in the figure legends. For comparison between three or more groups, one-way analysis of variance followed by Tukey's test was used.

## Author contributions

HK-S, SW, and EI performed experiments. HK-S and AK analyzed data. HK-S and AK designed experiments. HK-S and AK wrote the manuscript. All authors reviewed and corrected the manuscript.

### Conflict of interest statement

The authors declare that the research was conducted in the absence of any commercial or financial relationships that could be construed as a potential conflict of interest.
