# Supplementary material for: miRNA miR-21 Is Largely Dispensable for Intrathymic T-Cell Development
Source: Front Immunol. 2018 Nov 5;9:2497. doi: 10.3389/fimmu.2018.02497 (PMC6230590; doi:10.3389/fimmu.2018.02497)
Supplement: Supplementary file 1 [file Data_Sheet_1.pdf]

Figure S1

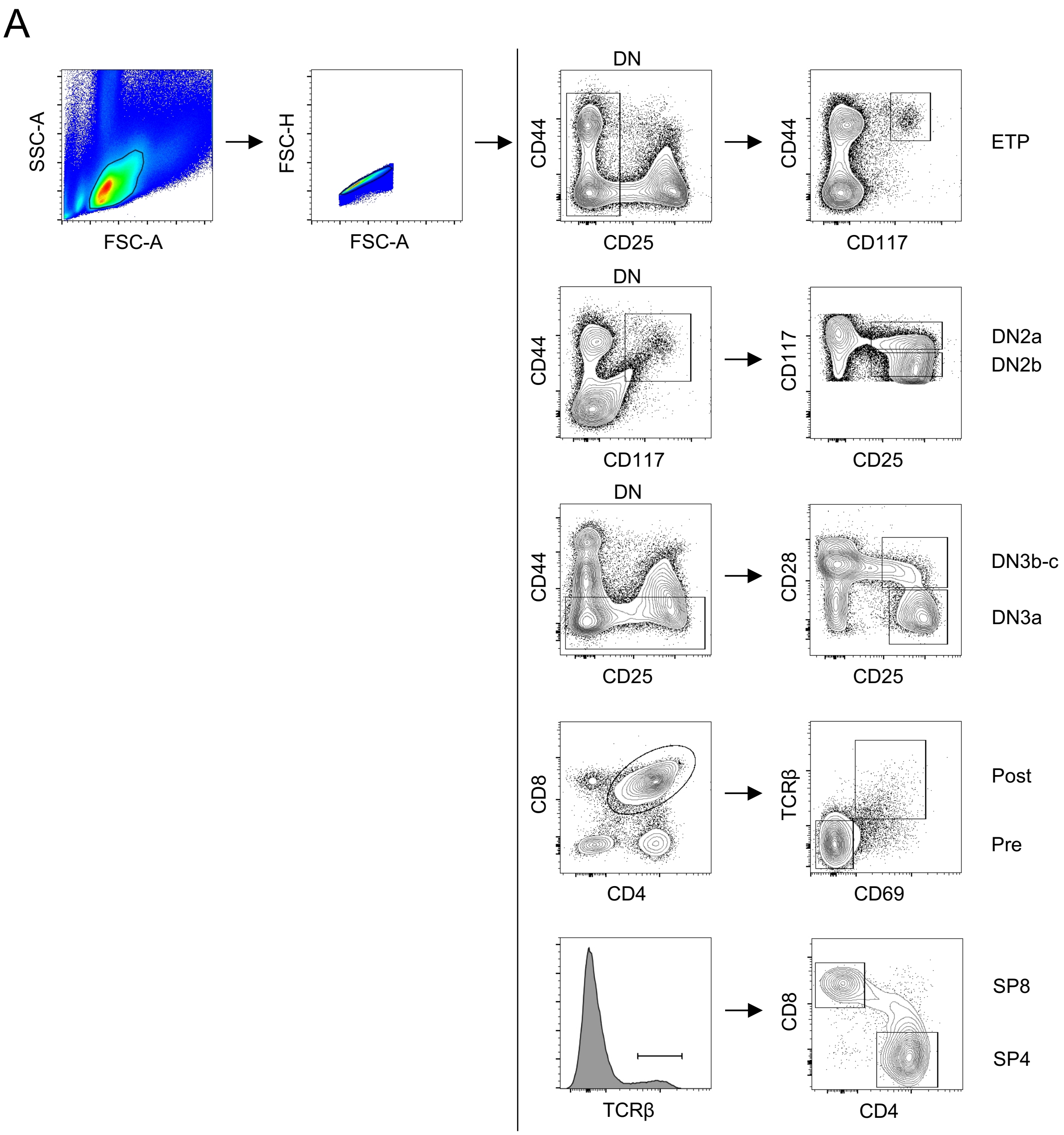

Figure S1. Gating strategy to identify T-cell subsets. A) Representative flow cytometric analysis to identify T-cell subsets of WT mice stained with antibodies against CD25, CD44, CD117, CD4, CD8, CD69, TCRβ. Subsets were defined as: ETPs ($CD117^{hi}CD25^{-}CD44^{+}$), DN2a ($CD117^{hi}CD25^{+}CD44^{+}$), DN2b ($CD117^{lo}CD25^{+}CD44^{+}$), DN3a ($CD25^{+}CD44^{-}CD28^{-}$) and DN3b-c ($CD25^{+}CD44^{-}CD28^{+}$), pre-selection DP ($CD4^{+}CD8^{+}CD69^{-}TCR\beta^{-}$), post-selection DP ($CD4^{+}CD8^{+}CD69^{+}TCR\beta^{+}$), SP4 ($TCR\beta^{+}$, $CD4^{+}$) and SP8 ($TCR\beta^{+}$, $CD8^{+}$).

Figure S2

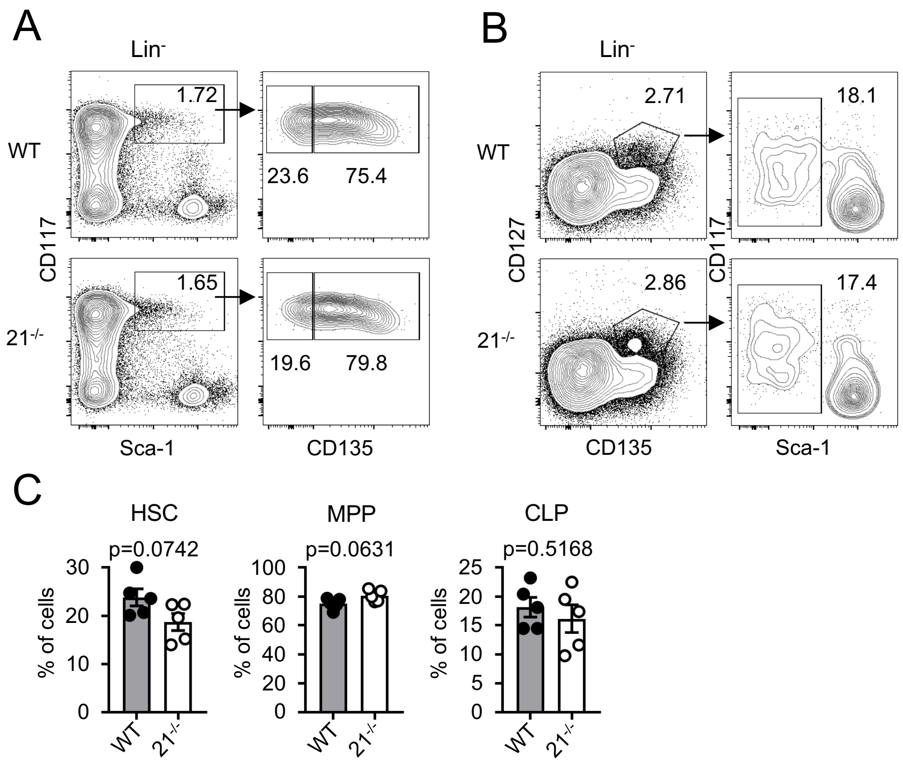

Figure S2. Characterization of BM-derived T-lineage progenitors in miR-21-deficient mice. A) Representative flow cytometric analysis of lineage-depleted BM from WT and miR-$21^{-/-}$ mice stained with antibodies against Sca-1, CD117 and CD135 to identify HSCs and MPPs. Numbers adjacent to gates represent frequencies relative to parent gate. **B)** Representative flow cytometric analysis of lineage-depleted BM from WT and miR-$21^{-/-}$ mice stained with antibodies against CD135, CD127, Sca-1 and CD117 to identify CLPs. Numbers adjacent to gates represent frequencies relative to parent gate. **C)** Statistical analysis of flow cytometric results shown in **A)** and **B)**. Each dot represents one mouse, n = 5 per genotype.

Figure S3

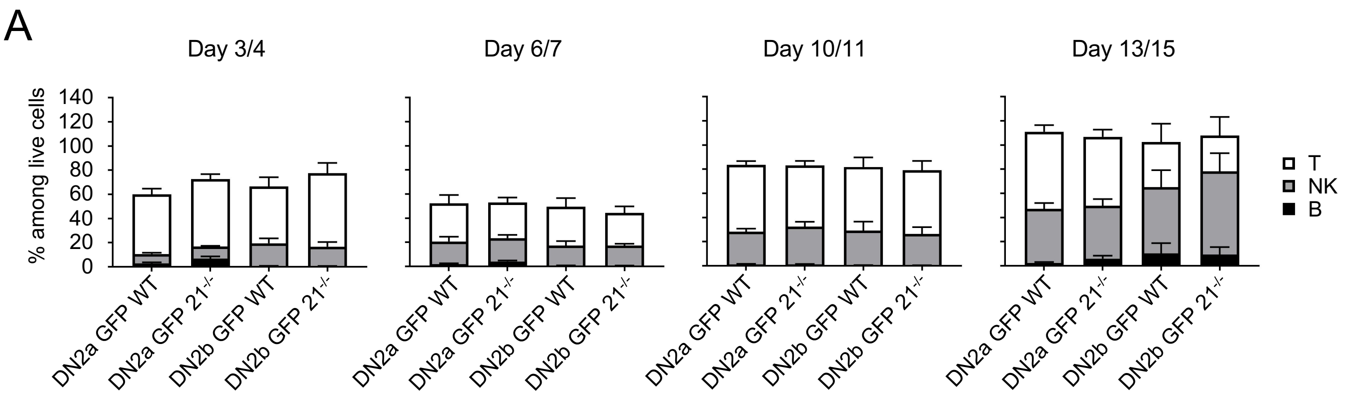

Figure S3. Alternative lineage fate decisions are not promoted in the absence of miR-21. A) Sorted DN2a and DN2b cells were cultured on OP9-GFP cells for up to 15 days. Generation of T, NK and B cells was assessed by flow cytometry at indicated periods of time. Bar graphs show pooled data from two independent experiment, *n* = 4-7 mice per group.

Figure S4

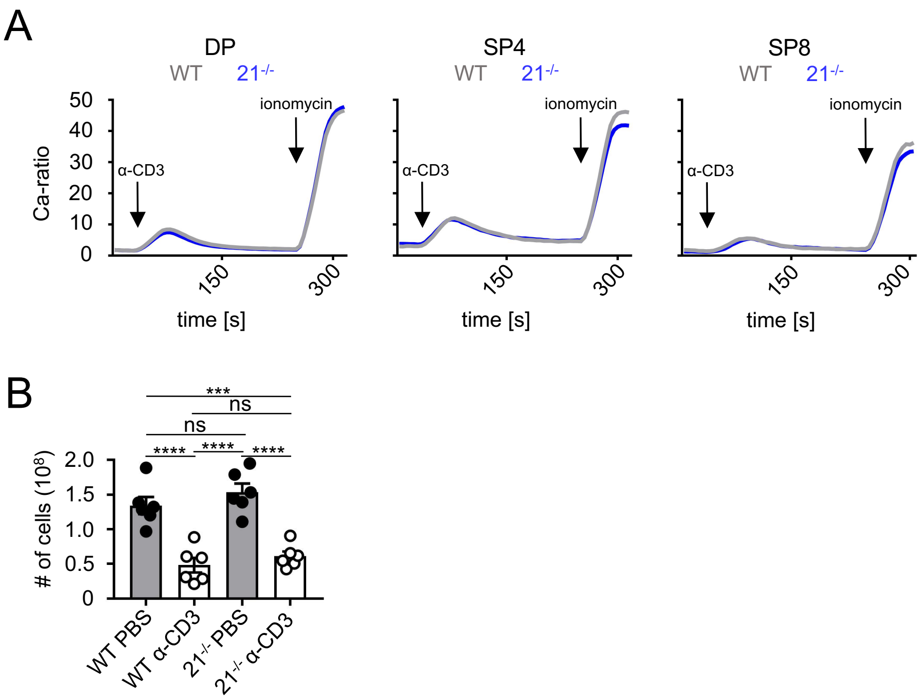

Figure S4. Negative selection is not altered in the absence of miR-21. A) Representative flow cytometric analysis of Ca-ratio over time of DP, SP4 and SP8 thymocytes stimulated with anti-CD3 and ionomycin as a control. **B)** Total cellularity of thymi from WT and miR-$21^{-/-}$ mice, 48 hours post injection with either PBS or α-CD3, n = 6 for each genotype. Pooled data of two independent experiments.

Figure S5

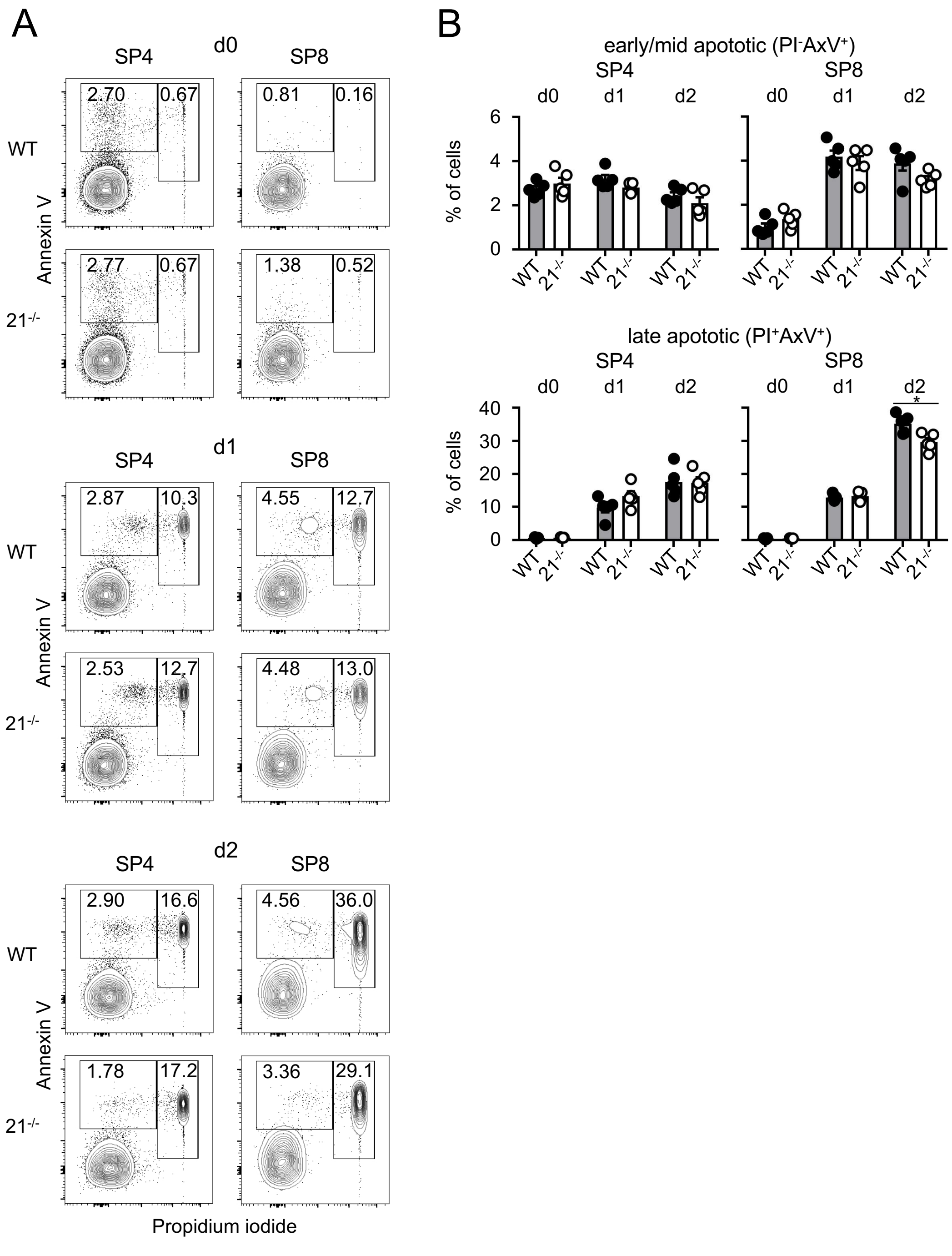

Figure S5. Apoptosis of SP T cells *in vitro* is not disrupted in miR-21-deficient mice. A) Representative flow cytometric analysis of thymi from WT and miR-21$^{-/-}$ mice stained with antibodies against CD4, CD8 as well as Propidium iodide (PI) and Annexin V (AxV). Numbers adjacent to gates represent frequencies of early/mid (defined as PI$^-$AxV$^+$) and late apoptotic cells (defined as PI$^+$AxV$^+$). Frequencies were determined at indicated days. **B)** Statistical analysis of flow cytometric results shown in **A)**. Each dot represents one mouse, $n = 5$ per genotype.
